# Botulinum Toxin Use for Modulating Neuroimmune Cutaneous Activity in Psoriasis

**DOI:** 10.3390/medicina58060813

**Published:** 2022-06-16

**Authors:** Marius Nicolae Popescu, Cristina Beiu, Mădălina Gabriela Iliescu, Mara Mădălina Mihai, Liliana Gabriela Popa, Ana Maria Alexandra Stănescu, Mihai Berteanu

**Affiliations:** 1 Department of Physical and Rehabilitation Medicine—“Elias” Emergency University Hospital, “Carol Davila” University of Medicine and Pharmacy, 020021 Bucharest, Romania; marius.popescu@umfcd.ro (M.N.P.); mberteanu@gmail.com (M.B.); 2 Department of Oncologic Dermatology—“Elias” Emergency University Hospital, “Carol Davila” University of Medicine and Pharmacy, 020021 Bucharest, Romania; mara.mihai@umfcd.ro (M.M.M.); liliana.popa@umfcd.ro (L.G.P.); 3 Department of Medical Rehabilitation, “Ovidius” University, 900527 Constanta, Romania; iliescumadalina@gmail.com; 4 Department of Family Medicine, “Carol Davila” University of Medicine and Pharmacy, 020021 Bucharest, Romania; alexandrazotta@yahoo.com

**Keywords:** botulinum toxin, psoriasis, neurogenic inflammation, neuropeptides, neuroimmune system

## Abstract

Psoriasis is a complex immune-mediated inflammatory disorder that generates enormous interest within the scientific communities worldwide, with new therapeutic targets being constantly identified and tested. Despite the numerous topical and systemic medications available for the treatment of psoriasis, alternative therapies are still needed for the optimal management of some patients who present with localized, resistant lesions. Novel insights into the contribution of cutaneous neurogenic inflammation in the pathogenesis of psoriasis have yielded exciting new potential roles of nerve-targeting treatments, namely botulinum toxin type A (BoNT-A), for the management of this disease. This paper aims to review the existing literature on knowledge regarding the potential role of BoNT-A in psoriasis treatment, with a focus on its ability to interfere with the immunopathogenetic aspects of psoriatic disease. Furthermore, in our paper, we are also including the first report of psoriatic lesions remission following local BoNT-A injections that were administered for treating upper limb spasticity, in a patient that concomitantly suffered from psoriasis and post-stroke spasticity.

## 1. Introduction

Psoriasis vulgaris is an immune-mediated chronic disease that presents with characteristic cutaneous inflammation. While the pathogenesis of this highly complex disease is still far from being fully elucidated, keratinocyte abnormal hyperproliferation and immune system dysfunctions are well recognized as pivotal contributors, with numerous innovative treatments targeting unique immunologic features of the psoriatic disease. Involvement of neurogenic inflammation in the pathogenesis of psoriasis is a less explored mechanism. It was first indicated in early studies that identified, within the psoriatic skin, an increased concentration of nerve fibers as well as a high level of neuropeptides of cutaneous sensory nerve origin, primarily calcitonin gene-related peptide (CGRP) and Substance P (SP) [1,2].

Furthermore, psoriatic lesions have demonstrated spontaneous clinical remission after local loss of innervation, or nerve injury [3,4,5,6,7]. Hence, it was postulated that cutaneous sensory afferent neurons are involved in the evolution of psoriasis plaques and may serve as a feasible therapeutic target for psoriasis [8,9].

Botulinum toxin (BoNT) is a potent neuromodulator, that serves as a highly versatile drug, with numerous clinical applications for both therapeutic and aesthetic purposes. The evolving knowledge of the role of BoNT in inhibiting neurogenic inflammation, mainly by impeding nerve-derived release of CGRP and SP, has contributed to its ‘off label’ use for the management of various inflammatory skin disorders, including psoriasis, rosacea [10], atopic dermatitis [11], alopecia [12,13,14], and many other dermatological conditions [15,16,17].

Rosacea is a disease characterized by both neurogenic inflammation and neurovascular hypersensitivity [18] and intradermal injections of BoNT into the upper dermis were shown to positively influence the function of free nerve endings and neurovascular complex [10]. Likewise, in the first placebo-controlled study to evaluate the effect of BoNT in patients with atopic dermatitis, it was found to have anti-inflammatory and antipruritic effects, mainly due to the ability to inhibit nerve-derived neurotransmitters, mast cell activation and eosinophil infiltration, as well as via modulation of local vasodilation [11]. Furthermore, BoNT was proved to aid hair regrowth in inflammatory conditions affecting the hair follicle, including cephalgia alopecia, alopecia areata, and androgenetic alopecia. In cephalgia alopecia, the proposed mechanism is believed to be the regulation of hair growth by inhibiting neuropeptides SP and CGRP release from the nerve fibers located around the hair follicle stem cells, since very high levels of SP can elicit an abnormal immune response that triggers hair follicles and causes hair loss [14]. SP has also been proven to be an important mediator of the inflammatory response in a mouse model for alopecia areata [19]. The exact mechanisms behind the effect of BoNT in androgenetic alopecia are still uncertain, and it has been hypothesized that BoNT scalp injections generate local muscle relaxation, and consequently alleviate microvascular pressure and enhance oxygen supply to hair follicles [13]. BoNT has also been administrated as an effective adjuvant treatment for various conditions that affect the intertriginous areas, such as Hailey–Hailey disease [20,21], Darier disease [22], and hidradenitis suppurativa [23,24]. These conditions usually occur in axillary and inguinal folds and are known to be exacerbated by local hyperhidrosis and bacterial colonization. Improvement after local BoNT-A treatment was mainly attributed to its ability to reduce sweat production and tissue maceration; thus, reducing potential contamination with pathogenic bacteria [17].

This review paper aims to analyze the most relevant published papers, experiments, and clinical trials concerning the potential utility of BoNT in the treatment of psoriasis. We are also reporting our recent observation of the clearance of psoriatic lesions on an upper limb that was treated with BoNT injections for the treatment of post-stroke spasticity. To our knowledge, this is the first published observation on this aspect.

## 2. Neuroimmune Pathways in the Pathogenesis of Psoriasis

Nervous system implication in the pathogenetic mechanisms of psoriasis is supported by numerous studies showing a substantial increase in the total number of nerve fibers [1] and neuropeptides in psoriatic lesions [25], as well as by research detecting clinical clearance of psoriasis lesions after local nerve injury [3,4,5,6,7].

CGRP and SP are neuropeptides of sensory nerve origin that can influence the fine regulation of immune cell functions and the inflammatory response in various inflammatory diseases, including psoriasis [25,26]. Immunohistochemical studies have shown that these neuropeptides present an increased level in psoriatic lesions [2], along with an upregulation of their corresponding receptors [27]. Furthermore, the plasma CGRP level in psoriasis patients was also detected to be higher when compared to healthy subjects [28].

Neuropeptides receptors such as neurokinin-1 receptor (NK1R) and Receptor activity modifying protein (RAMP) are expressed on T cells and antigen-presenting cells (such as monocytes and endothelial cells), and can influence the functions and immune responses of T helper type 17 (Th17) cells, which are known to play a critical role in the pathogenesis of psoriasis [29].

By binding to NK1R on T cells, SP can stimulate Th17 cell differentiation [30] and production of interleukin-17A cytokine (IL-17A). Il-17A is a key effector cytokine in psoriasis, by further inducing the release of other several proinflammatory cytokines (such as TNF-alpha), stimulating abnormal keratinocytes activation and proliferation, and promoting angiogenic effects [31]. NK1R activation on monocytes also leads to elevated expression of several interleukins (IL-1b, IL-6, and IL-23) that enhance production of Th17 cells [32]. It has also been confirmed that NK1R stimulation is necessary for adequate Ca^2+^ flux and Ca^2+^-dependent signaling to assist Th17 cell survival [33].

CGRP can also directly increase production of IL-17A, through activation of its receptor RAMP1 on Th17. Furthermore, it was shown that, in vivo, IL-17A production is significantly suppressed in RAMP1-deficient mice [34]. Additionally, CGRP can stimulate endothelial cells to release an increased amount of IL-6 and further bias the outcome of antigen presentation towards the Th17-pole, with increased levels of IL-17A [35].

This synergy between the nervous and the immune system in psoriasis created the background for the use of BoNT as a promising neuromodulator treatment for psoriasis, as will be discussed below.

## 3. Botulinum Toxin Biology

BoNT is an injectable neuromodulator produced by the bacterium *Clostridium botulinum*, a Gram-positive bacillus that causes botulism [36]. Currently, there are seven immunologically different serotypes of BoNT (A-G) and only two serotypes, type A and type B are approved for clinical use, with Botulinum toxin type A (BoNT-A) being the most commonly used in medical practice [37].

The clinical utility of BoNT-A originates from the ability to block muscular contraction by inhibiting neurotransmission between peripheral nerve cells and muscle fibers [38]. For the muscles to contract, the neurotransmitter acetylcholine (ACh) must be released from synaptic vesicles in the neurons into the neuromuscular junction from where it can further bind to receptors on muscle fibers. This is possible with the aid of a complex of three important proteins called SNARE proteins (soluble N-ethylmaleimide-sensitive factor attachment protein receptor), which is essential for the fusion of synaptic vesicle and nerve terminal membrane, resulting in release of ACh into the synaptic cleft, and further binding to muscle fibers. When BoNT-A enters a nerve cell it cleaves and prevents the SNARE proteins from forming the complex [38]. Therefore, ACh cannot be released from the neurons into the neuromuscular junction and muscle fibers fail to contract. This original indication of BoNT-A leads to its clinical utility in a variety of medical disorders characterized by muscular hyperactivity, including blepharospasm and hemifacial spasm [39], strabismus [40], post-stroke upper-limb or lower-limb spasticity [41], and focal and generalized dystonia [42]. Currently, BoNT-A is also widely used for multiple aesthetic concerns that can be alleviated with local muscle relaxation, such as dynamic facial rhytides, particularly in the upper face [43].

Subsequently, the unique effects on cholinergic synapses even beyond the motor nervous system and the ability to inject the toxin directly into the targeted zone, led to its utility in a wide range of medical fields, with many indications being currently under investigation. When injected at the dermo-subcutaneous level, BoNT-A reduces the ACh release from autonomic nerve terminals that innervate smooth muscle and eccrine glands. Due to the ability to reduce the hyperactive function of eccrine sweat glands, BoNT-A is used as a safe and effective method for improving primary focal hyperhidrosis of the axillae, palms, and feet [44,45].

Furthermore, it was proven that BoNT-A also inhibits the release of neurotransmitters (e.g., CGRP and SP) from the sensory motor neurons. Since CGRP and SP are two important neuropeptides in the pathophysiological process of cutaneous neurogenic inflammation, it was hypothesized that BoNT-A can potentially be used for treating several inflammatory skin diseases, including psoriasis [46,47,48].

**Figure 1:**
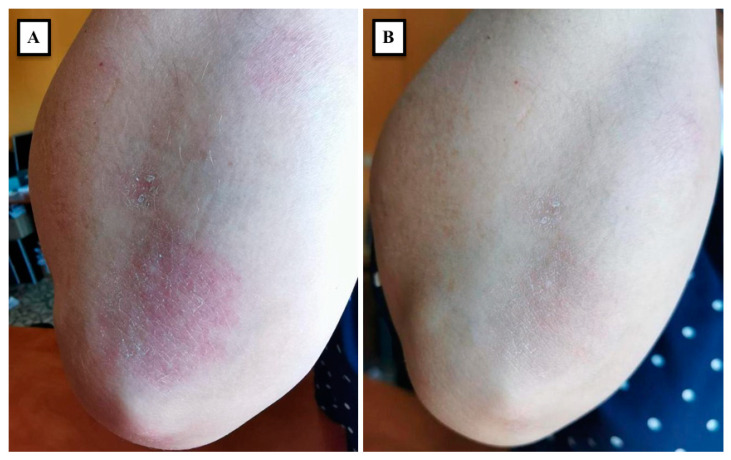
Psoriasis plaques on the extensor surfaces of the elbow and forearm before (**A**) and 1 month after (**B**) a single injection session with 1000 U of abobotulinumtoxinA for treating upper-limb spasticity.

The latter finding may represent the mechanistic basis to support our recent observation of the remission of psoriatic plaques following local BoNT-A injections that were administered for treating upper limb spasticity (Figure 1A,B). This was noticed in a 62-year-old female patient that was suffering from mild to moderate psoriasis for 14 years and had received multiple topical treatments in our dermatology department, namely emollients, corticosteroids, vitamin D analogs, and localized UVB phototherapy. Different combinations of these treatments led to major improvement, but complete clearance of the lesions was never observed. Figure 1A exhibits the maximum improvement the patient had obtained with the above-mentioned topical treatments: erythematous plaques, with a slightly scaly surface, were still present on the patient’s right elbow and forearm. After suffering a stroke, the patient developed upper limb spasticity and received local BoNT-A injections as part of her neurological rehabilitation program. A total dose of 1000 U of abobotulinumtoxinA (Dysport^®^, Ipsen Biopharm, Wrexham, UK) was delivered in a single injection session, under ultrasound guidance. The targeted muscles were the flexors of the elbow, wrist, and fingers. One month after, remission of the psoriatic lesions on the injected upper limb was noticed (Figure 1B). The patient stated that the symptoms of itching were also minimized. As observed in the clinical image, the patient suffered from a mainly inflammatory form of plaque psoriasis; thus, the outcome is in line with evidence pointing towards the effect of BoNT-A strictly on the inflammatory component of psoriasis [26]. Therefore, in our opinion, mainly hyperkeratotic psoriasis plaques may benefit from keratolytic topical therapy prior to BoNT-A local therapy, in an attempt to obtain similar clinical benefits.

## 4. Preclinical and Clinical Research

### 4.1. Preclinical Research

As pathogenesis of psoriasis is highly suspected to be partially modulated by skin innervation, a series of preclinical mouse studies have investigated the utility of BoNT-A in reducing cutaneous inflammation in psoriasis.

Using a transgenic KC-Tie2 psoriasiform mouse model, Nicole Ward demonstrated that surgical cutaneous denervation of peripheral nerves improves psoriatic skin inflammation, with a 30% improvement in acanthosis, a 30% decrease in CD4^+^ T cells, and a decrease of up to 40% in CD11c^+^ dendritic cells, concomitant with decreased IL-23 protein expression [49]. The effect was blunt when exogenous SP and CGRP were injected into the plaques of the murine model, suggesting that in psoriasis these neuropeptides promote acanthosis and support infiltration of immune cells into the skin; thus, mediating cutaneous neurogenic inflammation. This finding led to the hypothesis that chemical denervation could produce similar improvements and introduced that targeting nerve–immunocyte interactions should be investigated as potential psoriasis therapeutic treatment strategies.

Thus, in another experimental study using the same KC-Tie2 mouse model of psoriasis, Ward et al. investigated the effect of intradermal injection of BoNT-A (9 units/kg diluted in 1 mL saline) versus saline injection [48]. At 2 and 6 weeks after treatment, histological and immunostaining analyses were performed and showed improvements in psoriasis activity similar to those obtained with surgical denervation, namely lymphocytic infiltration and acanthosis were markedly reduced compared to the control site.

### 4.2. Clinical Research

Preclinical findings demonstrating the efficacy of intradermal injections of BoNT-A in improving psoriasis skin lesions were successfully translated to the treatment of several patients with recalcitrant psoriasis. Our literature research with the terms “psoriasis” and “botulinum toxin” led to the identification of 3 clinical trials and 3 case reports. Table 1 schematizes the papers reviewed.

In a small clinical open-label trial, Zanchi et al. evaluated the efficacy of BoNT-A for the treatment of 15 patients suffering from inverse psoriasis [50]. A single injection of onabotulinumtoxinA (a total dosage of 50–100 units per patient, depending on the extent and severity of the affected sites) was injected intradermally across the psoriatic lesions. At 12 weeks’ follow-up, all 15 patients showed improvements in subjective symptoms (pain and itch), and 13 patients showed objective reduction in erythema extension, intensity, and infiltration. The authors hypothesized the effect was primarily obtained by targeting the neuroglandular junction to reduce local sweating, skin maceration, and secondary infection in the affected region. The ability of BoNT-A to inhibit the release of local pro-inflammatory neuropeptides was also considered to be an important contributory factor. Similarly, Saber et al. reported a case in which 100 units (U) of onabotulinumtoxinA was successfully used to treat inverse psoriasis and concomitant hyperhidrosis in the axillae [51]. Again, the authors hypothesized that BoNT-A can improve inverse psoriatic lesions related to hyperhidrosis either by the reduction in hyperhidrosis and further reduction in the bacterial/yeast colonization that are known as aggravating cofactors in inverse psoriasis, or even by exerting a direct effect on psoriasis lesions.

AbobotulinumtoxinA also proved efficacy in treating psoriatic lesions. By injecting a total of 30 U of abobotulinumtoxinA directly into a recalcitrant psoriatic plaque, Erin Gilbert and Nicole L. Ward reported major improvements in the lesion within 3 weeks post-injection, followed by a complete remission of the plaque, an effect that was sustained for up to 7 months [52].

Other authors have also quantified the effectiveness of neuromodulatory treatment of plaque psoriasis with onabotulinumtoxinA by using objective assessments for scoring psoriasis. A single-center pilot study conducted by Aschenbeck et al. showed that onabotulinumtoxinA treatment significantly alleviated the Psoriasis Area and Severity Index (PASI) and the Physician Global Assessment (PGA) scores in patients with recalcitrant, localized, psoriatic plaques [53]. Punch biopsy specimens obtained from lesional skin 2 weeks prior and 8 weeks after treatment were immunostained and showed reduction in the expression of neuropeptides SP and CGRP, and an increase in epidermal nerve fibers (ENFs) density. Recently, in a descriptive cross-sectional study with 8 patients, Gonzalez et al. evaluated the improvement in psoriasis plaques following a single dose of abobotulinumtoxinA injections, by measuring the Total Clinical Score (TCS) composed of the following parameters: sum of erythema (0–3), desquamation (0–3), and infiltration (0–3) of each psoriasis plaque. At week 4, a major improvement of 38.5% of the initial TCS score was recorded (*p* = 0.001) [54].

Unfortunately, the first randomized double-blinded clinical trial (BoNT-A vs. vehicle) of single-dose BoNT-A injections in patients with plaque psoriasis did not show serious effectiveness versus placebo. Ten patients were randomized for BoNT-A as single therapy (nine injections of 4 U, 0.04 mL in total) or sodium chloride therapy as the control group. The trial conducted by Todberg et al. failed to demonstrate any clinical or histological differences from the vehicle. However, the authors concluded that even if the research failed to find an effect of a single-dose BoNT-A injection on psoriasis plaques, it is unknown if more repeated doses could impact results more positively [55].

Nevertheless, Botsali et al. were the first to explore the utility of abobotulinumtoxinA local injections for managing nail psoriasis, in two patients that suffered from severe nail psoriasis, with extensive involvement of both the nail bed and nail matrix. Two main strategies were employed: BoNT-A injections as a monotherapy in one patient, and BoNT-A injections as an adjuvant to systemic methotrexate therapy, in the second patient—who had previously failed to respond to multiple conventional systemic therapies for psoriasis. After a single injection of abobotulinumtoxinA (30 U per affected nail in patient 1 and 15 U per affected nail in patient 2), delivered under ring-block anesthesia, both patients achieved highly satisfactory improvements [56].

## 5. Conclusions

In conclusion, nerve-targeting treatment may represent an underexplored therapy area in psoriasis. Based on the published literature reviewed in this paper, BoNT-A neuromodulator may constitute a novel useful adjunct for the treatment of recalcitrant plaque psoriasis. We are also reporting, for the first time, the remission of psoriatic lesions following local BoNT-A injections that were administered for treating upper limb spasticity, in a patient that concomitantly suffered from psoriasis and post-stroke spasticity. Further randomized controlled studies are needed for clarifying the reasons behind our observation and for determining the efficacy and safety of BoNT-A for the local treatment of psoriasis.

## Figures and Tables

**Table 1:** Representative clinical trials and case reports on the utility of botulinum toxin type A (BoNT-A) in localized psoriasis treatment.

First Author [Ref.], Year	Study Design	Number of Patients Included	Affected Sites	Type and Doses of BoNT-A injected	Follow-Up	Results
Zanchi [50], 2008	Single-arm (BoNT-A) pilot study	15	Inverse psoriasis: axillae (7), inframammary folds (6)*,* intergluteal sulcus (7), groin skin folds (5), and umbilicus (1)	OnabotulinumtoxinA: 50–100 U per patient	12 weeks	VAS score for itching and pain improved in all patients, and 87% of patients showed reduction in skin erythema and infiltration
Saber [51], 2011	Case report	1	Inverse psoriasis: axillary regions	OnabotulinumtoxinA: 100 U per axilla	1 month	From large brightly erythematous well-demarcated axillary plaques to only minimal residual erythema
Gilbert [52], 2014	Case report	1	Left buttock	AbobotulinumtoxinA: 30 U	7 months	Total remission of the injected plaque was achieved, and the effect was maintained for 7 months post-treatment; local relapse was noticed at 8 months post-injection
Aschenbeck [53], 2018	Single-center pilot study	8	Elbow (4), back (1), knee (1), leg (1), foot (1)	OnabotulinumtoxinA: 53 U in average (range, 25–98 U)	10 weeks	Clinically, PASI and PGA score were significantly decreased; Immunohistochemically, increased ENF density and decreased expression of the neuropeptides SP and CGRP were detected 8 weeks after the injection
Gonzalez [54], 2020	Descriptive cross-sectional study	8	Specific sites were not reported (12 plaques included in total)	AbobotulinumtoxinA: 5 U per cm2 of lesional skin (a maximum of 50 U)	4 weeks	Average TCS showed statistically significant clinical improvement; two patients reported a significant reduction in pruritus
Todberg [55], 2018	Randomized double-blinded trial	10	Specific sites were not reported (up to two target lesions were selected per patient)	AbobotulinumtoxinA: 36 U for a psoriatic plaque	8 Weeks	No clinical or histopathological statistically significant differences between BoNT-A and placebo groups
Botsali [56], 2020	Case report	2	Nail psoriasis	AbobotulinumtoxinA: Patient 1: 30 U per affected nail; Patient 2: 15 U per affected nail	Patient 1: 8 months; Patient 2: 6 months	Patient 1: VAS score decreased from 9 to 3 Patient 2: VAS score decreased from 6 to 2 on the 4th month, and was consistent with 3 on month 6 post-injection

VAS: Visual Analogue Scale; PASI: Psoriasis Area and Severity Index; PGA: Physician’s Global Assessment; ENF: Epidermal Nerve Fibers; SP: Substance P; CGRP: Calcitonin Gene Related Peptide; TCS: Total Clinical Score.

## Data Availability

Not applicable.
